# Maintaining the quality of postharvest broccoli by inhibiting ethylene accumulation using diacetyl

**DOI:** 10.3389/fnut.2022.1055651

**Published:** 2022-11-15

**Authors:** Xiaotong Li, Zan Meng, Aman Ullah Malik, Song Zhang, Qingguo Wang

**Affiliations:** ^1^College of Food Science and Engineering, Shandong Agricultural University, Taian, China; ^2^Postharvest Research and Training Centre, Institute of Horticultural Sciences, University of Agriculture Faisalabad, Faisalabad, Pakistan

**Keywords:** broccoli, yellowing, ethylene biosynthesis, maintain quality, diacetyl

## Abstract

Broccoli (*Brassica oleracea* L. var. *Italic*) is rich in nutrition. However, it is susceptible to yellowing after harvest, leading to nutritional and economic losses. In this study, diacetyl, a natural food additive compound, was selected to inhibit the yellowing of broccoli florets and maintain the nutrient quality during storage time. It was found that 20 μl L^–1^ diacetyl treatment for 12 h could significantly delay the yellowing and decrease the weight loss and lignin content of broccoli florets. Meanwhile, diacetyl could maintain higher contents of chlorophyll, vitamin C and flavonoids and suppress the transcript levels of chlorophyll degradation–related genes in broccoli florets. Moreover, accumulations of reactive oxygen species (ROS) were inhibited by diacetyl treatment. Under diacetyl treatment, the generation of ethylene was prevented by inhibiting the activities and related-gene expressions of 1-aminocyclopropane-1-carboxylic acid (ACC) synthase and ACC oxidase. Based on our findings, exogenous diacetyl could be employed as a novel bioactive molecule for retarding the yellowing and maintaining the quality of postharvest broccoli.

## Introduction

Broccoli (*Brassica oleracea* L. var. *Italic*) is favored by consumers as it is rich in vitamin C, soluble fibers, and nutraceutical compounds (1). However, it tends to rapidly senesce after harvest. Senescence often causes quality deterioration, such as yellowing, water loss, increasing in lignin content, and decreasing of vitamin C, flavonoids, and other nutrients, eventually leading to losses of the commercial value (2).

Several studies have been devoted to investigating the mechanism of broccoli senescence. Ethylene was reported to play an essential role in regulating the postharvest quality of broccoli (3). Wounding could induce ethylene synthase in broccoli florets (4). 1-Aminocyclopropane-1-carboxylic acid (ACC) synthase (ACS) and ACC oxidase (ACO) were crucial enzymes in the ethylene synthesis pathway, which played a critical role in regulating the quality of broccoli (5). Lowering the gene expression and activities of *ACS* and *ACO* could retard the floret yellowing of broccoli (6). The inhibition of *BoACO2* expression in broccoli decreased the biosynthesis of ethylene and kept it green. Previous studies showed that the *BoACS1*, *BoACS2* and *BoACS3*, which encoded ACC synthase, were differentially expressed in the senescence course of broccoli florets (7). Meanwhile, reactive oxygen species (ROS) were also sharply accumulated by harvesting broccoli and up-regulated by ethylene (8, 9). Excessive ROS accumulations induced by biotic or abiotic stresses caused oxidative stress damage to broccoli, which accelerated chlorophyll degradation and then led to the loss of quality. The antioxidant system of plant was gradually enhanced to resist oxidative stress with the senescence processing (10, 11). In previous study, the broccoli displayed yellowing during senescence with the decreasing chlorophyll content, which was triggered by ROS and ethylene (11). Thus, lower ethylene and ROS contents were beneficial to maintaining the broccoli quality.

Series of enzymes were reported to regulate the chlorophyll degradation. Firstly, chlorophyll degradation is the transformation of chlorophyll *b* into chlorophyll *a*, which is catalyzed by CBR (chlorophyll *b* reductase) and HCAR (7-hydroxymethyl chlorophyll *a* reductase) (2). The CBR enzyme is encoded by the genes of *NYC1* (*NON-YELLOW COLORING 1*) and *NOL* (*NYC-LIKE*) (12). During the early chlorophyll-degrading process, it is confirmed that pheophytinase (PPH, encoded by *PPHs* genes) took part in removing pheophytin and forming pheophorbide. Then pheophorbide *a* oxygenase (PAO, encoded by *PAO* gene) accelerates the unfolding of the porphyrin macrocycle, which further promotes the chlorophyll degradation (13). Moreover, *SGR* (Stay Green) gene, the upstream of the PAO pathway, was also involved in the degradation of chlorophyll (14).

Various physical and chemical techniques have been used to maintain the quality of broccoli. Physical techniques include low temperature (15), modified atmosphere packaging (16), ultraviolet irradiation (17), and so on. But these methods require a huge expense. Some chemical compound was also used in postharvest quality maintenance. For example, 1-methylcyclopropene (1-MCP) is a chemical agent for retarding yellowing of broccoli effectively (18). Although some chemosynthetic preservatives are effective and the residue is low enough, the food safety issue is still concerned by consumers. Some natural food additives are reported to be used for preserving fruit and vegetables. For example, chitosan oligosaccharides can alleviate the calyx senescence of mandarin fruits by decreasing the abscisic acid content (19, 20). Folic acid can inhibit the senescence of broccoli by improving the antioxidant capacity (21). Therefore, the development of safe and natural preservatives is the hotspot of future research (22).

Diacetyl, also known as 2,3-butanedione, is naturally present in bay leaves, honey, wine, and balsamic vinegar (23, 24). It was also an aroma component of ripe fruit, such as jalapeno peppers, sweet peppers (25), and lucuma (*Pouteria lucuma*) fruit (26). The US Food and Drug Administration (FDA) believes that the ingestion of diacetyl in food is generally recognized as safe (GRAS)^1^. Diacetyl is also a widely used food additive in China (GB1886.51-2015). Recent studies have shown that diacetyl can act as an anti-microbial organic composition applied in mandarins, grapes, apples and strawberries (27, 28). Moreover, diacetyl can inhibit abiotic stress-induced senescence in *Arabidopsis* according to the latest study (29). It implies that diacetyl can be used in vegetables senescence during storage. Little information is available on the influence of diacetyl on the postharvest quality of fruit and vegetables during storage.

The objective of this study was to study the effect of diacetyl on inhibiting the yellowing of broccoli florets and maintaining the nutrient quality during storage time. Thus, the postharvest broccoli florets were treated with various concentrations of diacetyl during storage time.

## Materials and methods

### Chemicals

Diacetyl (PubChem CID:650) was obtained from Macklin Biochemical Co., Ltd. (Shanghai, China).

### Plant material and treatments

Broccoli (*Brassica oleracea* L. var. Italica, cv. You-xiu) was purchased from Aolaifeng Market, Tai’an, Shandong, China, and transported to our laboratory as soon as possible. The broccoli heads with tight florets and uniform size, maturity, color, and free from diseases and mechanical damage were chosen for the subsequent study. The selected broccoli heads were washed with tap water, drained, and dried with paper towel. All operations of measurement and fumigation with diacetyl were carried out in the ventilation equipment to ensure that the experimental operators were not exposed to the volatile.

Experiment 1 was carried out to investigate the effect of diacetyl on the visual quality of broccoli florets and determine the suitable concentration to inhibit the senescence. The broccoli heads were cut carefully with a sharp knife into florets with length of 7–8 cm and approximately 15 g each. Then the florets were randomly divided into six groups, about 350 g per group. The selected 350 g broccoli florets as one replicate (three replicates for each concentration) were put in a container with a total volume of 5 L (LocknLock Co., Ltd.), sealed, and then fumigated at 25 ± 1°C for 12 h at concentrations of 0, 1, 5, 10, 20, and 40 μl L^–1^ (volume of liquid diacetyl/volume of the container) diacetyl. The concentration in the control group was 0 μl L^–1^. After fumigation, the container was opened, ventilated, and then covered with the lid again but not sealed. The florets were kept at 25 ± 1°C for 4 d. The changes in quality were evaluated visually and recorded using images daily.

Experiment 2 was designed to investigate the effect of diacetyl on the objective qualities of florets and explore the mechanism. Twenty-one boxes of broccoli florets were prepared and fumigated in the same way as in experiment 1 but with only 0 and 20 μl L^–1^ (the selected optimum concentration of diacetyl from Expt. 1). At shelf life of 0, 1, 2, 3, and 4 d after fumigation, broccoli florets were randomly selected every day from each treatment and three independent biological replicates were set. After measuring the color changes, chlorophyll content and ethylene production for each floret, the remaining florets were frozen with liquid nitrogen, ground into powder, and finally stored at –80°C for further physiology and biochemical analysis.

### Color changes and chlorophyll content

The color of broccoli florets was assayed using the method proposed by Xu et al. (21). It was measured with a digital colorimeter (CR-400, Konica Minolta, Japan) and the a* (red, +or green, −), b* (yellow, +or blue, −) values were determined daily. The sampled broccoli florets from each replicate were randomly selected and tested at 5 equidistant points. The contents of chlorophyll *a* and *b* were determined according to the method of Sun et al. (30). Three samples of the fresh broccoli florets were collected at each time point (0 d, 1 d, 2 d, 3 d, 4 d), and 0.5 g of fresh broccoli florets were taken. Then 30 ml of ethanol (95%) was added to extract for 22 h (normal temperature and avoid light). The resulting supernatant was collected and used as a blank control. The absorbance at 470 nm, 665 nm, and 649 nm wavelength was measured to calculate the concentrations of chlorophyll *a* and *b* using the following equation:

Ca = 13.95 × A665nm-6.88 × A649nm

Cb = 24.96 × A649nm-7.32 × A665nm

Ca and Cb are the concentrations of chlorophyll *a* and *b*, respectively.

### Weight loss, vitamin C, flavonoid, and lignin contents

The weight loss was assayed as described by Xu et al. (21).

The content of vitamin C was measured according to the method reported by Sohail et al. (31). Frozen broccoli floret powder (10 g) was extracted with 20 ml of *meta*-phosphoric acid–acetic acid solution. Then 5 ml of solution was added to 2 ml of the ground extract, and the mixture was filtered through a cheesecloth. Samples were titrated in 2,6-dichloroindophenol dye solution until a light pink color developed and kept for 5 s. The content of vitamin C was represented as g kg^–1^ of broccoli.

Flavonoid content was determined according to the NaNO_2_–Al (NO_3_)_3_ colorimetric method (32). Frozen broccoli floret powder (1 g) was mixed with 30 ml of 70 % ethanol, extracted for 1.5 h at 65°C, and then centrifuged at 10,000 × *g* for 20 min at 25°C. The supernatant extract (1 ml) was added to 70 % ethanol (1 ml) and 0.3 ml of 5 % NaNO_2_, mixed thoroughly and placed for 6 min. Then 0.3 ml of 10 % Al (NO_3_)_3_ was added to the mixture. The mixture was placed at 25°C for 6 min. Subsequently, 2 ml of 4 % NaOH was added and reacted for 10 min. The absorbance was measured at 510 nm. Rutin was used as a standard to calculate the flavonoid content. The concentration of flavonoid was represented as g kg^–1^.

The lignin content was determined as described by Yu et al. (33). Frozen broccoli floret powder (2 g) was weighed, added to a 15-ml centrifuge tube containing 5 ml of precooled 95 % ethanol, and centrifuged at 10,000 × *g* for 15 min. The sediment was washed with 95 % ethanol and ethanol–hexane, collected, and completely dried at 60°C. The dried sediment was mixed with 1 ml of 25 % acetylacetonate in acetic acid and reacted at 70°C for 30 min. Then 1 ml of 2 mol L^–1^ NaOH was added to the aforementioned mixture to end the reaction, followed by the addition of 0.1 ml of 7.5 mol L^–1^ hydroxylamine hydrochloride and 2 ml of glacial acetic acid, and centrifugation at 12,000 × *g* for 10 min. The absorbance of supernatant was measured at 280 nm. The lignin content was represented as g kg^–1^ based on fresh weight.

### Ethylene generation rate, ACS enzyme activity, and ACO enzyme activity

The ethylene generation rate was determined according to Zaharah et al. (34). One hundred gram of fresh broccoli florets were sealed in a 5-L container and stored at 25°C for 12 h. Furthermore, 1 ml of headspace gas was injected into a gas chromatography (7820A, Agilent Technologies, Inc., the United States of America). The ethylene level was calculated according to the linear relationship between the peak area and ethylene concentration.

#### 1-aminocyclopropane-1-carboxylic acid synthase activity

The ACC synthase activity was measured according to the method described by Zaharah et al. (34). Briefly, 10 ml of extraction buffer (containing 1 mmol L^–1^ ethylene diamine tetraacetic acid (EDTA), 1 mmol L^–1^ phenylmethylsulfonyl fluoride (PMSF), 4 mmol L^–1^ dithiothreitol (DTT), 3 % polyvinylpolypyrrolidone (PVPP), and 10 μmol L^–1^ pyridoxal phosphate) was mixed with 2 g of frozen powder of broccoli flower buds, swirled and shocked immediately, and then centrifuged at 4°C and 12,000 × *g* for 30 min. The supernatant was collected to obtain the enzyme extract.

The enzyme extract (0.5 ml) and reaction buffer (1.5 ml) were added to a 10-ml sample bottle (with a rubber stopper) and placed at 30°C for 1 h. Then 0.1 ml of 25 mmol L^–1^ HgCl_2_ solution was injected to terminate the reaction, and the mixture was placed in an ice bath for 10 min. Subsequently, 0.2 ml of precooled 5 % NaClO–saturated NaOH solution was added, shaken quickly for 5 s, and incubated in the ice bath for 5 min. The gas (1 ml) was extracted from the headspace, and the amount of ethylene generation was determined by gas chromatography.

#### 1-aminocyclopropane-1-carboxylic acid oxidase activity

1-aminocyclopropane-1-carboxylic acid oxidase activity was measured according to the protocol of Zaharah et al. (34). A volume of 10 ml of extraction buffer (containing 10% glycerin, 5% PVPP, 5 mmol L^–1^ DTT, 30 mmol L^–1^ sodium ascorbate, and 0.1 mmol L^–1^ FeSO_4_) was mixed with 2 g of frozen powder of broccoli flower buds. The remaining measurement steps were the same as that in Section 2.5.1.

The enzyme extract (0.5 ml) and reaction buffer (1.5 ml) were added to a 20-ml sample bottle (with a rubber stopper). Subsequently, 1 ml of NaHCO_3_ was injected into the sample bottle and incubated at 30°C for 30 min. Then 1 ml of the gas was extracted from the headspace to determine the ethylene release.

### Hydrogen peroxide (H_2_O_2_) content, superoxide anion (O_2_^–^) content, peroxidase (POD) enzyme activity and catalase (CAT) enzyme activity

The contents of H_2_O_2_ and O_2_^–^ were assayed according to the protocols of Hu et al. (35) and Jin et al. (36).

The activities of CAT and POD were determined based on the method proposed by Hu et al. (35). One gram of frozen broccoli powder was added to 5 ml of 0.1 mol L^–1^ PBS (pH = 7) containing 0.05 g PVPP, swirled and shocked immediately and centrifuged at 4 °C and 12,000 × g for 15 min, the supernatant was used to analyze the enzyme activity. The CAT activity was assayed by recording the decrease every 30 s for 3 min at 240 nm, the reaction system consisted of 2 ml of PBS, 0.8 ml of 0.3 % H_2_O_2_ and 0.5 ml of supernatant. The POD activity was measured according to the oxidation of guaiacol by hydrogen peroxide. The reaction liquid included 50 ml of PBS, 19 μL of 30 % H_2_O_2_ and 28 μl of guaiacol. The absorbance value of the mixture was recorded every 30 s for 3 min at 470 nm. The results were expressed in U kg^–1^.

### Ribonucleic acid extraction and RT-qPCR analysis

Total RNA of broccoli florets was extracted using an Omini Plant RNA Kit (Cowin Biosciences, Beijing, China) and cDNA was obtained using a HifiScript cDNA Synthesis Kit (Cowin Biosciences, Beijing, China), respectively, following the manufacturers’ instructions. The concentrations of total RNA and cDNA were determined using the BioPhotometer D30 (Eppendorf AG, Germany).

The primers of RT-qPCR used in this study were reported in published articles (2, 5, 37–39). All primers used (Supplementary Table 1) in this study were synthesized by Sangon Biotech Co., Ltd. (Shanghai, China). The Ultra SYBR Mixture Kit (Cowin Biosciences, Beijing, China) was used in RT-qPCR assays.

### Statistical analyses

In this study, all experimental designs were fully performed with three biological replicates. The SPSS software was used to conduct the significant difference by the least significant difference test (*P* < 0.05, *P* < 0.01). The data were expressed as means ± standard deviations.

## Results

### Effects of diacetyl treatment on yellowing of broccoli florets during shelf life

**FIGURE 1:**
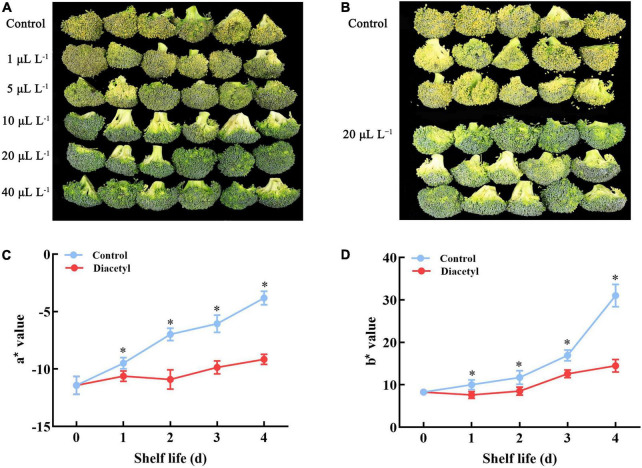
Diacetyl delayed broccoli floret yellowing during shelf life. Appearance of broccoli florets treated with different concentrations of diacetyl **(A)**, appearance of broccoli florets treated with 20 μl L^–1^ diacetyl **(B)**, a* **(C)** and b* **(D)** values of broccoli florets treated with 20 μl L^–1^ diacetyl. Data are the average of three replicates ± SD. Asterisk (*) indicates a significant difference among the control and diacetyl treatment groups at the same time at *P* < 0.05.

The broccoli florets gradually lost green color and then decayed, leading to the development of off-odors. On day 4, the florets of the control obviously yellowed and decayed. Whereas, 5, 10, 20, and 40 μl L^–1^ diacetyl treatments maintained greener color and better quality compared with the control. The results showed that the anti-yellowing effect was increased with higher concentrations of diacetyl (Figure 1A). Thus, we selected 20 μl L^–1^ diacetyl treatment for the further research (Figures 1B–D). It was found that a* and b* values of broccoli florets showed an increasing trend in four days (Figures 1C,D). The b* values of diacetyl treatment were lower than that in control, indicating a decreased yellowing degree with diacetyl treatment. The a* value of the control increased obviously, while the value of the florets under diacetyl treatment changed a little during shelf life and was lower than the control at the end of four days. The values of a* and b* (Figures 1C,D) were consistent with the visual color (Figure 1B). Our study suggested that suitable concentrations of diacetyl treatment could maintain the visual quality of broccoli during ambient storage.

### Effects of diacetyl treatment on broccoli florets quality during shelf life

**FIGURE 2:**
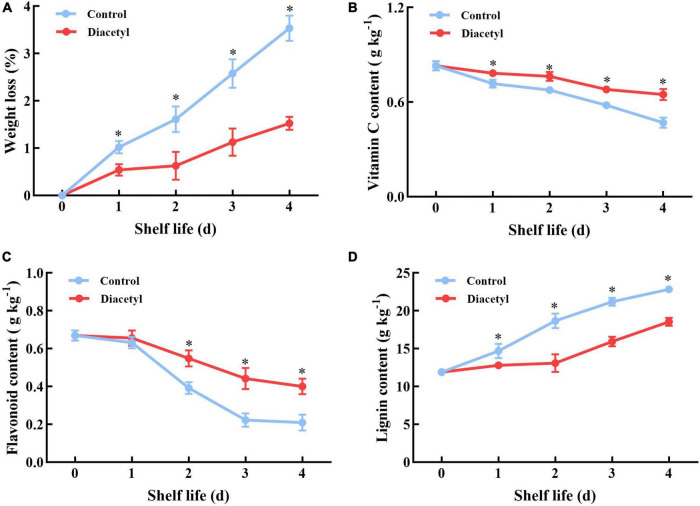
Diacetyl reduced the weight loss **(A)** and inhibited the decrease in the contents of vitamin C **(B)** and flavonoid **(C)** and the increase in the lignin content **(D)** in broccoli florets during shelf life. Data are the average of three replicates ± SD. Asterisk (*) indicates a significant difference among the control and diacetyl treatment groups at the same time at *P* < 0.05.

Then the quality of broccoli florets was studied during shelf life. Firstly, weight loss of broccoli florets was measured with or without the diacetyl treatment. It was found that broccoli florets showed a slower weight loss under diacetyl treatment compared to the control (Figure 2A). Broccoli is rich in vitamin C and flavonoid (2), thus the contents of vitamin C and flavonoid were also analyzed. As demonstrated in Figures 2B,C, diacetyl-treated florets showed higher contents of vitamin C and flavonoid than the control during shelf life. Meanwhile, texture is one of the important quality indices of fruit and vegetables. The lignin content is closely correlated with textural changes. As a component of the cell wall, the lignin content can reflect the senescence degree (40). According to Figure 2D, accumulation of lignin increased slowly in the diacetyl-treated broccoli florets. Therefore, these results suggested that diacetyl treatment could maintain the quality of broccoli florets during shelf life.

### Effects of diacetyl treatment on chlorophyll degradation of broccoli florets during shelf life

**FIGURE 3:**
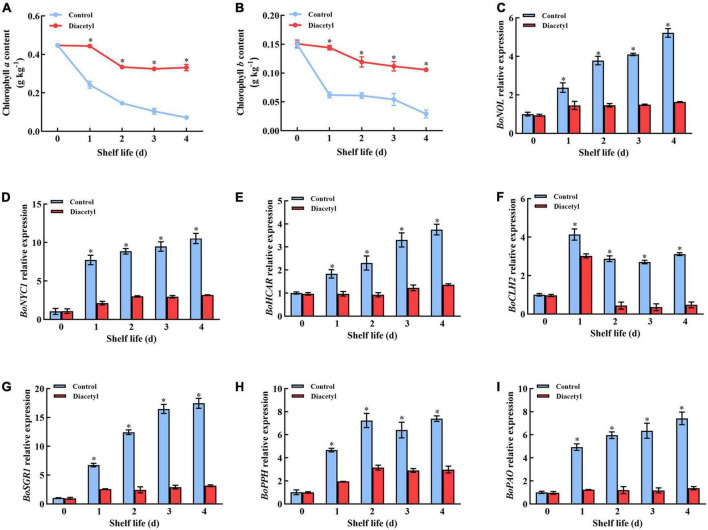
Diacetyl treatment inhibited the decrease in the chlorophyll content and the expression of chlorophyll degradation–related genes broccoli florets during shelf life. Chlorophyll *a* content **(A)**, chlorophyll *b* content **(B)**, and the relative expression level of *BoNOL* **(C)**, *BoNYC1* **(D)**, *BoHCAR* **(E)**, *BoCLH2* **(F)**, *BoSGR1* **(G)**, *BoPPH* **(H)**, and *BoPAO* **(I)**. Data are the average of three replicates ± SD. Asterisk (*) indicates a significant difference among the control and diacetyl treatment groups at the same time at *P* < 0.05.

The levels of chlorophyll *a* and *b* presented an overall downward trend during broccoli florets shelf life, whereas diacetyl-treated plants maintained a higher chlorophyll content than the control (Figures 3A,B). It indicated that diacetyl could inhibit the degradation of chlorophyll in broccoli florets. These results were consistent with the differences in a* and b* values between the control and treated florets.

Then various chlorophyll degradation-related genes were selected to perform further exploration. The transcript levels of *BoNOL*, *BoNYC1*, *BoHCAR*, *BoCLH2*, *BoSGR1*, *BoPPH*, and *BoPAO* genes showed an upward trend during shelf life in both control and treated broccoli florets (Figures 3C–I). However, the transcript levels of all these genes were always lower after diacetyl treatment. The results were consistent with the chlorophyll content (Figures 3A,B). It suggested that diacetyl inhibited chlorophyll degradation by suppressing the expression of chlorophyll degradation-related genes in broccoli florets.

### Diacetyl treatment could inhibit senescence-associated genes through repressing reactive oxygen species accumulations in broccoli florets

**FIGURE 4:**
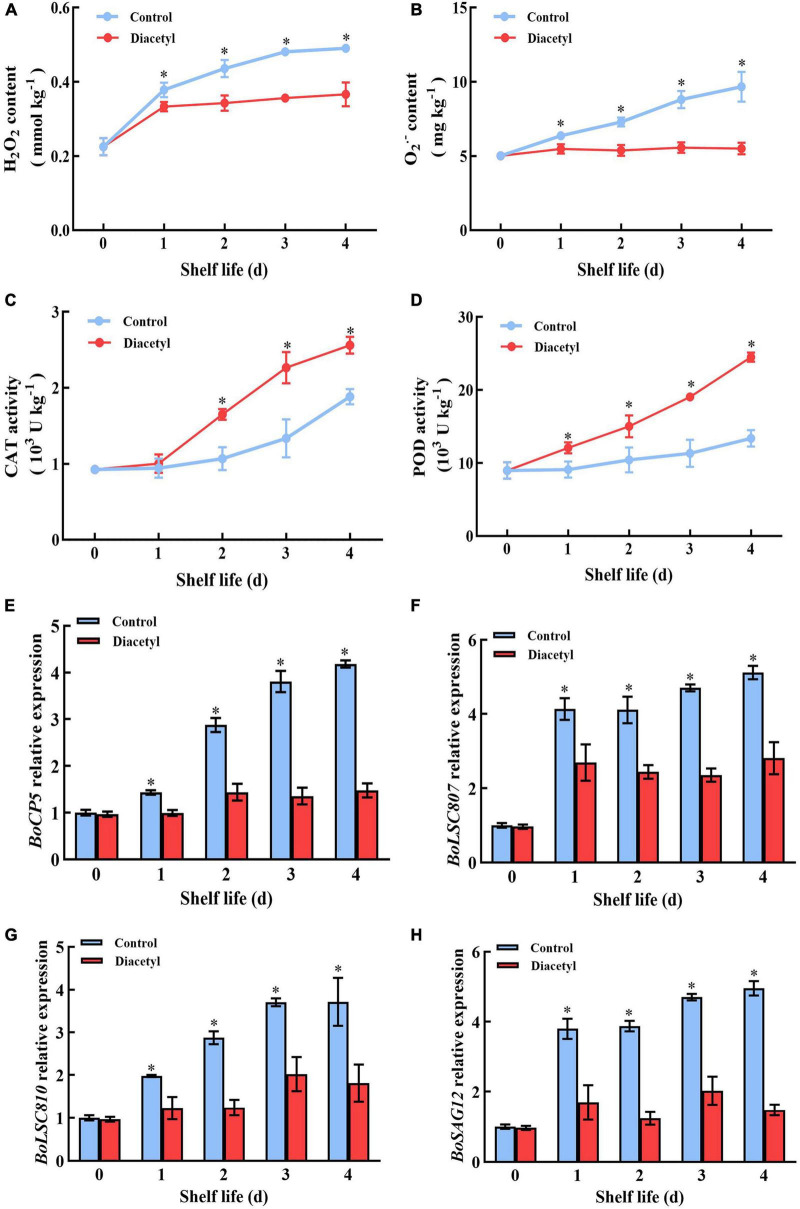
Diacetyl treatment inhibited the accumulation of ROS and the transcription level of senescence-associated genes in broccoli florets. H_2_O_2_ content **(A)**, O_2_^–^ content **(B)** and the enzyme activities of POD **(C)** and CAT **(D)**. Relative expression of *BoCP5* **(E)**, *BoLSC807* **(F)**, *BoLSC810* **(G)** and *BoSAG12* **(H)**. Data are the average of three replicates ± SD. Asterisk (*) indicates a significant difference among the control and diacetyl treatment groups at the same time at *P* < 0.05.

The accumulations of ROS showed increasing trends in broccoli over time. Whereas, the levels of H_2_O_2_ and O_2_^–^ were lower than that in control with the diacetyl treatment (Figures 4A,B). Then the activities of POD and CAT were determined. It was found that the activities of POD and CAT were higher in diacetyl treated broccoli, although all showed an overall increasing trend (Figures 4C,D). It suggested that diacetyl treatment could improve the antioxidant capacity and scavenge the accumulation of ROS during shelf life.

Excessive accumulations of ROS could aggravate the expression of senescence associated genes (41, 42). Thus, the transcript levels of several broccoli senescence-marker genes, including *BoCP5, BoLSC807, BoLSC810 and BoSAG12* (5, 43), were detected. All of them were induced in the control groups during shelf life (Figures 4E–H), while these senescence-associated genes did not show significant difference with those in the diacetyl-treated group. These results exhibited that diacetyl could inhibit the postharvest senescence of broccoli through decreasing ROS accumulation.

### Diacetyl treatment inhibit ethylene generation in broccoli florets during shelf life

**FIGURE 5:**
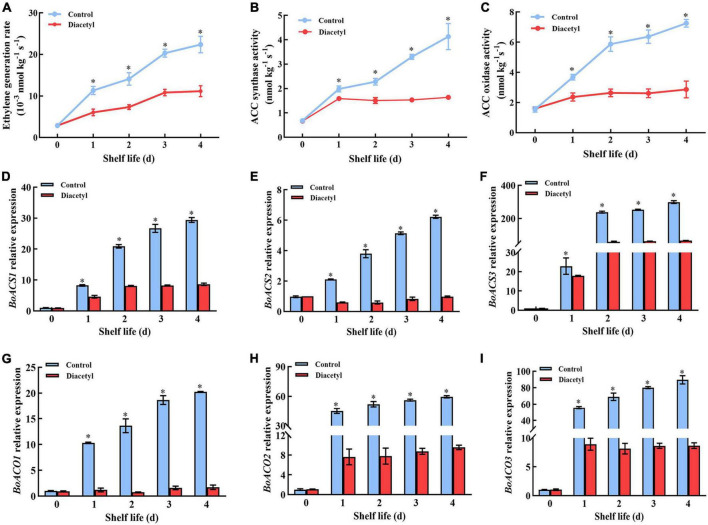
Diacetyl treatment inhibited the ethylene generation of broccoli florets during shelf life. Ethylene generation rate **(A),** ACS activity **(B)**, ACO activity **(C)** and the transcription levels of *BoACS1* **(D)**, *BoACS2* **(E)**, *BoACS3* **(F)**, *BoACO1* **(G)**, *BoACO2* **(H)**, and *BoACO3* **(I)**. Data are the average of three replicates ± SD. Asterisk (*) indicates a significant difference among the control and diacetyl treatment groups at *P* < 0.05.

It was reported that ethylene played an essential role in regulating the senescence of broccoli during postharvest (3). Thus, the contents of ethylene were measured after diacetyl treatment. It showed that the ethylene generation rate in the control was much higher than that in the treated florets (Figure 5A). This result indicated that diacetyl could inhibit the generation of ethylene in broccoli florets. Then the activities of ACS and ACO were detected in broccoli. The ACS and ACO activities under diacetyl treatment were lower than that in the control after 1 d (Figures 5B,C). As shown in Figures 5D-I, the gene expression of *BoACO1*, *BoACO2*, *BoACO3 BoACS1*, *BoACS2*, and *BoACS3* was also obviously suppressed by diacetyl treatment. These results demonstrated that lower ethylene generation in broccoli florets with diacetyl treatment could be ascribed to lower *ACS* and *ACO* transcript levels and enzyme activities. Therefore, suppressing ethylene production is crucial to maintaining broccoli quality by diacetyl treatment.

## Discussion

After harvest, the disruption of water and nutrient supply to broccoli heads can boost senescence, resulting in chlorophyll degradation and loss of nutritional value (21). The harvested broccoli florets usually turn yellow in two days at 25°C. Our study indicated that diacetyl could inhibit the yellowing of broccoli florets (Figure 1) and delayed the nutritional loss of vitamin C, flavonoid and the generation of lignin at suitable concentrations (Figure 2). Excessive ROS accumulation could accelerate the quality loss of broccoli. Scavenging ROS overproduction and maintaining ROS homeostasis were considered as a strategy to maintain the quality of postharvest broccoli. For example, 24-epibrassinolide was able to alleviate yellowing of broccoli by enhancing the antioxidant capacity (11). In this study, diacetyl treatment improved the enzyme activities of POD and CAT to prevent the contents of H_2_O_2_ and O_2_^–^. Therefore, diacetyl treatment alleviated yellowing of broccoli via improving the antioxidant capacity and maintaining the ROS equilibrium.

Broccoli florets faded at the beginning of yellowing because of the degradation of chlorophyll (21). Some genes, including *BoCLH2*, *BoPPH*, *BoPAO*, *BoNYC1*, *BoNOL*, *BoHCAR* and *BoSGR1*, have been reported to be involved in the regulation of chlorophyll degradation during broccoli senescence (37, 44). Previous studies showed that ethylene induced the expression of *BoPPH and SGR1*, which were accompanied by the yellowing of broccoli (44). Likewise, the gene expression of *BoPPH* and *BoSGR1* were induced by broccoli senescence in this study. However, it was suppressed after diacetyl treatment, which could be relevant to the suppression of ethylene production. In our research, the expressions of *BoNYC1* and *BoNOL*, which strikingly increased in untreated broccoli florets, were inhibited by diacetyl treatment during shelf life. Thus, the transcript levels of *BoNYC1* and *BoNOL* were attenuated by diacetyl to retard chlorophyll degradation. HCAR could interact with other chlorophyll catabolic enzymes, such as SGR1/NYE1, NYC1 and NOL, during leaf senescence in *Arabidopsis* (45). Meanwhile, Jara et al. (2) suggested that HCAR played a protective role due to its contribution to the stability of photosystem II, which prevented the release of chlorophyll. Thus, diacetyl could inhibit the expression of *BoHCAR*, which might be associated with *BoSGR1*, *BoNYC1*, and *BoNOL*.

Previous studies concluded that ethylene accumulation was relevant to the quality loss of broccoli. Ethanol vapor and phytosulfokine α treatment could inhibit the senescence of broccoli by repressing the ethylene synthesis-related genes’ transcription and enzyme activities (6, 46). Similarly, the diacetyl treatment reduced the activities of ACS and ACO through inhibiting the expression of ethylene synthesis genes, such as *BoACS1*, *BoACS2*, *BoACS3*, *BoACO1*, *BoACO2*, and *BoACO3*, thereby further reducing ethylene generation in this study (Figure 5). Moreover, some studies have shown that ethylene could regulate the transcription levels of genes encoding major chlorophyll degradation and ROS metabolism enzymes (47, 48). For example, ethylene insensitive 3 (EIN3), a positive regulator of ethylene signaling, accelerated chlorophyll degradation by physically binding to *NOL*, *NYC1* and *PAO* promoters to induce their expression. Moreover, ORE1 was a direct target of EIN3, and was induced by ethylene. It could also activate the expression of *ACS2* and subsequently promote the ethylene production (49, 50). Thus, we demonstrated that diacetyl treatment suppressed the synthesis of ethylene and then inhibited ROS accumulation, ultimately repressed the transcription level of chlorophyll degradation gene (Figures 3-5). We emphasized that the suppression of ethylene generation by diacetyl treatment was the principal factor for delaying the quality loss of broccoli.

Diacetyl, which naturally exists in some foods, is popular as a food-flavoring additive (24). According to centuries of human acquaint with diacetyl in fermented foods, FDA believes that there is no apparent health concerns about diacetyl used in food (see text footnote 1). The daily intake of diacetyl is 3,300 μg/person per day in Europe and 8,000 μg/person per day in the United States (World Health Organization). The diacetyl content in yogurt is 200–3,000 mg kg^–1^ (51). The dose of diacetyl used in this study was 20 μl L^–1^, about 281 mg kg^–1^ fresh broccoli, and the residual value of diacetyl in broccoli must be lower than the used dose. Therefore, the residual dose in broccoli should be safe for human beings. In summary, diacetyl has the potential to be used for maintaining the quality of broccoli based on its efficacy and the availability of safe management measures during and after fumigation treatment.

Overall, our study established that diacetyl decreased the postharvest quality decline, suppressed the degradation of chlorophyll, and improved the antioxidant capacity in broccoli, which was accompanied by lower ethylene generation. Several studies illustrated that ethylene had complex interactions with abscisic acid (ABA) and jasmonic acid (JA) during plant senescence (9). However, it is still unclear whether diacetyl could regulate the ethylene synthesis through ABA and JA in our research. Meanwhile, some questions need to be further explored. such as “how diacetyl affected the expression of ethylene synthesis–related genes,” “whether exogenous diacetyl treatment could stimulate endogenous resistance to stress factors and enhance the anti-senescence ability of plants” and “whether exogenous diacetyl can act as a signaling molecule to stimulate endogenous diacetyl and then enhance the anti-senescence ability of plants.” Based on our studies, we believed that diacetyl could be a novel and valuable molecular tool to explore the mechanism of senescence of postharvest fruit and vegetables in future.

## Conclusion

Appropriate concentrations of diacetyl could inhibit the yellowing and maintain the nutritional quality of broccoli florets. Diacetyl treatment prevented the excessive accumulation of ROS and improved the antioxidant capacity. Furthermore, diacetyl treatment suppressed the expression of ethylene synthesis–related genes, decreased the ethylene synthesis–related enzyme activities, which led to reduced ethylene generation. Therefore, diacetyl treatment could be considered as a meaningful strategy for alleviating senescence and maintaining the quality of postharvest broccoli, it also has a potential to be used as a molecular tool to explore plant senescence.

## Data availability statement

The original contributions presented in this study are included in the article/Supplementary material, further inquiries can be directed to the corresponding authors.

## Author contributions

XL: investigation, formal analysis, visualization, writing—original draft, and writing—review and editing. ZM: formal analysis and writing—review and editing. AM and SZ: writing—review and editing. QW: project administration, supervision, conceptualization, funding acquisition, resources, and writing—review and editing. All authors contributed to the article and approved the submitted version.

## Footnotes

1
https://www.fda.gov/food/food-additives-petitions/food-additive-status-list
