# Supplementary material for: Maintaining the quality of postharvest broccoli by inhibiting ethylene accumulation using diacetyl
Source: Front Nutr. 2022 Nov 15;9:1055651. doi: 10.3389/fnut.2022.1055651 (PMC9707704; doi:10.3389/fnut.2022.1055651)
Supplement: Supplementary file 1 [file Data_Sheet_1.PDF]

Supplementary data:

Supplementary Table 1 Primers for qRT-PCR

Gene name	Forward primer sequences (5′-3′)	Reverse primer sequences (5′-3′)
**Actin**	CCAGAGGTCTTGTTCCAGCCATC	GTTCCACCACTGAGCACAATGTTAC
**BoCLH2**	ATCTTTTAACAGTAGGCTTATCATC	GAATAAAATGAGTTGTAGAGAAGGT
**BoPPH**	GAATTAGTGTGGCAAAAGATAAGTG	CATGAGACATATAGGAACACTGTTT
**BoPAO**	TATCTGATCCTTCCCATATCGATTT	ATGAGCAAATCCATATAACCCATTT
**BoNYC**	AGATACAATAGGGTTTATGTGCATC	TCATCCTTAGTTAAGAACCCTAACT
**BoNOL**	ATCTATGAAGCCGACTTACA	GATCTACTGCGATATCTACTCT
**BoHCAR**	TGGAGCAGCATCTTAATCT	AAGTTGGACCTTGTAATCTTG
**BoSGR1**	GATTTTCGAATCATCCAAGTTGAAA	CTGAGAATTGTTAATGGAGTGAGAA
**BoACS1**	TAACTTGGTTTTGATTTGGTTTCAG	GAACTTAACCAAAGATTTCAAGCAA
**BoACS2**	GGCGAACTGGGGTTGAGATT	GTCGTGCCCAAAGGGTTAGA
**BoACS3**	GACTTGAGGTGGCGTACAGG	AGCGGATTTGATGGGTTGGT
**BoACO1**	CGACTCCTTTAGCAAACAAATTTAT	ACTTGAAAGATAAACCGTTGATAGT
**BoACO2**	TTTAGATACTCTGGAGACTGAAGTT	ATATGTGTTTTAGGGCTGAATCTTT
**BoACO3**	TTTAGATACTCTGGAGACTGAAGTT	ATATGTGTTTTAGGGCTGAATCTTT
